# Trends in Delirium Rate Across 14 Hospitals During the COVID-19 Pandemic: A Comparative Study.

**DOI:** 10.1093/geroni/igab046.2715

**Published:** 2021-12-17

**Authors:** Ariba Khan, Marianne Klumph, Alexander Schwank, Michael Malone, Asma Sabih

**Affiliations:** 1 Advocate Aurora Health Care, Milwaukee, Wisconsin, United States; 2 Advocate Aurora Health, Milwaukee, Wisconsin, United States

## Abstract

During the COVID-19 pandemic, strategies to prevent delirium in the hospital were limited due to restrictions in staff and visitor policies. Thus, we suspected the delirium rate may increase during the pandemic. This study aimed to investigate the trends in delirium rate over past 2-years and compare this trend prior-to-and-during the COVID-19 pandemic in hospitalized older adults. Data was retrospectively obtained from the Acute-Care-for-Elders Tracker snapshot, an electronic health record tool to identify the presence of delirium within 48hrs of hospitalization for patients ≥65 years. Periods of interests were 3/2019-6/2019 (pre-COVID) and 3/2020-6/2020 (during-COVID). A weighted rate was calculated for each month by combining data from all hospitals for the total number of inpatients ≥65 years. The overall trend in the delirium rate was assessed with simple linear regression models and an ANCOVA. A χ2 and a Wilcoxon-Signed-Rank-Test were utilized to test for differences in the overall delirium rate between two time periods. Overall median delirium rate was 6.8% in 70,562 encounters of 42,878 patients (mean age= 78 years; mean length-of-stay= 6.5 days). The median delirium rate increased by 2.1% (6.6%to8.6%), for pre-COVID vs. during-COVID, respectively (Z=-3.044,p<0.001). There were no significant differences between actual and projected weighted delirium rates (p=0.18). However, the weighted delirium rate—for both the actual and projected trend lines—demonstrated significant changes over time (p<0.001).The trend in delirium rate increased over the study time period regardless of the pandemic. Further analyses with longer time-frame are crucial to understand the consequences of the pandemic on delirium rate.

